# Acute Inflammation of the Serous Membranes of the Brain and Spinal Cord; Described from Personal Observation at the Bedside

**Published:** 1846-10

**Authors:** 


### ART. VIII.

*Die Acute Entzündung der Serösen Häute der Gehirns und Rückenmarks. Nach eigenen Beobachtungen am Krankenbett geschrieben.* Von Dr. JOSEPH NEISSER, Prakt. Arzt zu Berlin.—*Berlin,* 1845.

*Acute Inflammation of the Serous Membranes of the Brain and Spinal Cord; described from Personal Observation at the Bedside.* By Dr. JOSEPH NEISSER, Physician at Berlin.—*Berlin,* 1845. 8vo, pp. 454.

AN apology to our readers is, we think, scarcely necessary for introducing to them a practical work on inflammatory affections of the brain. A correct diagnosis and treatment have an important social as well as a technical value. Insanity is considered by many to be a greater evil than death; imprisonment in a lunatics' hospital more distressing than entombment. We are convinced that a more correct diagnosis and treatment in this class of cases, would not only prevent the supervention of that form of chronic cerebral disease which constitutes insanity, but would obviate the serious mistake sometimes made by practitioners in the removal of cases of acute arachnitis to lunatic asylums as cases of mania. We know of more than one family on which the stigma of hereditary tendency to insanity has thus been fixed, and, we believe, we know also cases which have been maltreated in consequence of this mistake.

Dr. Neisser's work is divided into three parts: the first part, termed prolegomena, is a general consideration of the pathology of serous inflammation; the second is a detailed account of seven cases of cerebral and spinal disease; the third (the epilegomena) is an essay on the pathology and therapeutics of acute affections of the cerebro-spinal axis.

The history of these cases is, we think, peculiarly instructive. The symptoms as they arose from day to day are considered in their pathological, physiological, and therapeutical relations; the prognosis, diagnosis, and treatment are carefully deduced from a detailed review of previous experience or empirical knowledge, and from physiological data; and the whole forms both a useful contribution to medical art and a valuable criticism on medical science. It is, in short, an excellent monograph on the class of diseases investigated and treated by the author.

The serous membranes have little physiological interest; their function is only of a secondary character; but when attacked by disease they become of immense importance from the great influence they exercise over the contained organ. Inflammations of the serous membranes within the thorax, for example, are amongst the most common and, at the same time the most dangerous diseases; they are also much more frequently the seat of inflammation than the subjacent organs, if we except the lungs; they are justly considered by the practitioner as the most important class of inflammatory affections that he has to treat; for, independently of the primary diseases in organs to which they give rise, they are fruitful in secondary or sympathetic affections.

The structure of serous membranes is compound: they consist of a substratum of cellular membrane, well supplied with blood-vessels, and an unvascular epithelium. The substratum of cellular membrane is made up of several layers of fibrous tissue, the fibres of which cross each other, and have a regular arrangement. Whenever the vessels of the substratum are congested, the unvascular epithelium is raised, and projects into the cavity which it invests. The superficial distribution of the vessels of serous membranes renders the latter liable to rapid inflammation when the former are congested; and facilitates effusion through the parietes of the former into the serous sac.

The external surface of a serous membrane is attached to the organs or muscles external to the sac by the subserous cellular tissue; its anatomical peculiarities differ according to the anatomical relations of the true serous membranes, and the peculiarities give rise to characteristic pathological differences. The more plentifully the parts to which this subserous cellular tissue is attached are supplied with blood-vessels, the more is the serous membrane itself predisposed to inflammation, on account of the free anastomosis which takes place between the vessels of the contiguous structures. Thus the pulmonary pleura exhibits the most intense inflammatory action at the roots of the lungs, where the great vessels and bronchi enter; and at the fissure between the pulmonary lobes, where there is a layer of loose vascular tissue beneath the pleura. For the same reason, inflammation of the arachnoid membrane is more frequent on that side in connexion with the vascular pia mater than on that next to the dura mater.

A somewhat similar anatomical relation to the connecting subserous cellular tissue determines whether the inflammation be limited to the membranes or be extended to the organ it invests. Pulmonary pleuritis is rarely confined to the pulmonary pleura, and generally implicates the lungs, because its subserous cellular tissue is in immediate relation with the cellular tissue which connects the air-vesicles. Inflammation of serous membranes in direct connexion with fibrous tissues less frequently implicates the subjacent organs, because although the fibrous tissue is formed out of cellular membrane, it is comparatively less vascular.

The first stages of serous inflammation are the same as in other organs. Irritation being applied, the capillaries first contract, and so retard the circulation through them; then they are dilated and congested. Consequent on this is an effusion of serum through the parietes of the vessels, and the first result of serous inflammation is produced. The unyielding nature of the membrane itself facilitates the effusion as the dilatation of the capillaries is thereby restricted. If the inflammation ceases spontaneously, or is checked by art, the effused serum is readily taken up by the lymphatics; but if the inflammatory action continues, or increases in intensity, the blood-globules are heaped up in the capillaries, lose their colouring matter, and change their form; the blood then begins to coagulate within the capillaries; the lymphatics are no longer capable of performing their office, and the first stage of the disease, or the stage of inflammatory exudation, is accomplished.

The character of the exudation differs, however, with the progress of the disease. At first, when the capillaries are but slightly dilated, it is only watery serum; then plastic lymph or fibrin is effused, then pus; for the coats of the vessels become thinner and more porous as distension of them increases.

The products of serous inflammation differ in amount and character, from various circumstances which we need not enumerate, as these are well stated by systematic writers, and no doubt well known to our readers. False membranes are much less usual in the cerebral arachnoid sac than in the pleural or peritoneal; they are seldom, if ever, seen as a result of spinal arachnitis. Here the plastic power is at its minimum; in the serous membrane covering the heart and lining the pericardium it is at its maximum; the two being often strongly and intimately united by organized fibrin. The most remarkable exudation is that in which the colouring matter of the blood is effused, and sometimes even the blood itself. This variety is rare; it has been observed in inflammation of all serous membranes, but most frequently in that of the pericardium. Its true pathology is not yet expounded; but it indicates an intense and rapidly fatal disease.

Dr. Neisser discusses the pathology of serous inflammation as elucidated by microscopic researches, and especially the pathology of false membranes. The question of their formation is intimately connected with the physiology of development, and especially with the new doctrine of development by cells. There can be no doubt that researches of this kind will lead to new views both in pathology and treatment. What is the reason that the false membranes on mucous surfaces, as, for example, those formed in croup and in dysentery, never exhibit traces of organization? Why is the tendency to the formation of organized membranes so great in pericarditis, so insignificant in arachnitis? Dr. Neisser also takes occasion to discuss the question whether there be really a vis medicatrix, and resolves it in the negative: the processes developed in disease differ in no respect from those by virtue of which the embryo is developed and the tissues repaired. In reality, these processes lead to death; as, for example, in pericarditis and pleuritis, in fatal cases of which we often find the so-called healing process has established a close and intimate union between the contiguous membranes; constituting in fact one of the most incurable results of serous inflammation.

Dr. Neisser discusses at length the semeiology, etiology, and complications of serous inflammation; he is evidently intimately acquainted with his theme, and makes many interesting remarks. We do not, however, propose to enter into this subject at present, and we shall therefore proceed at once to a notice of the cases. The first shall be introduced in our author's own words as an example of clinical inquiry.

"First case. *Thursday,* 16.7.40 [*July* 16*th,* 1840]. Gotthold, of young and vigorous constitution, aged 20 years, was received yesterday into the inner division of the Charité hospital at Berlin, in an unconscious state. We have no further information respecting him or his previous condition. The patient lies on his back, his head turned towards the right side, with closed insensible eyes, very red and turgid cheeks, and almost altogether unconscious. Respiration unequal and irregular,—a couple of short and superficial expirations, then a deep sighing inspiration; considerable feverish heat; quick and full pulse—104 powerful strokes. (?)

"*Hemorrhagia cerebri, apoplexia sanguinea, complicated with inflammation and fever.*

"I. *Exegesis of the symptoms;* absolute significance of the symptoms of the functional anomalies considered alone. The first glance at the morbid condition leads to an indefinite opinion: the seat of the disease is within the cranium. The untaught readily distinguish cerebral diseases: he lies helpless there, his eyes closed, &c. We approach him, and investigate his condition; he mutters, but scarcely moves. There is sensual and mental disturbance.

"The disturbance of the intellect presents a passive form; he is insensible, and appears neither to hear nor see; no loud delirium, no excitement; no violent movements. Left to himself he remains apathetic; he speaks not, nor does he move. If questions be addressed to him, he certainly exhibits some tokens of consciousness, but these consist in a stammering of unintelligible words, which only demonstrate his delirium. There is a lively agitated movement of the right hand and foot to be seen occasionally, but the left extremities are never used. That is very significant; in fact, when the left hand is raised up, it falls mechanically as if by its own weight. If the *left* arm is pinched, he moves the right hand as if to prevent it. There is clearly also hemiplegia of the left side, and (as often happens) sensation is less impaired than motion. No reflex (involuntary) movements are excited by touching or pinching the affected limbs. The temperature is equally increased on both sides of the body. The countenance is unaffected, is not drawn. The eyes when opened are in the same axis, but insensible to light; the pupils are unaffected. No deviation of the point of the tongue, either to the right or left. He does not swallow what is given him to drink, —a circumstance indicative of a dangerous degree of seizure.

"The respiration is affected in a remarkable manner; at the first glance we hear a slight sighing and groaning of the patient, as if from great grief.

"On a more minute examination we find it as follows: the patient makes 1, 2, 3 respirations, which follow each other at equal intervals, but are quite superficial; we see only the first pair of ribs slowly rise and fall. Then, however, there occurs a full, deep inspiration, with distinct expansion of the thorax, and very often accompanied by a sigh. Again, 1, 2, 3, 4 superficial inspirations, interrupted by a deep and loud one, and so on. At the same time, it may be observed, that the left half of the thorax remains behind the right, and that the movements are effected almost altogether by the latter. Further, respiration inæqualis, unequal in the two halves; very abnormal in rhythm (respiratio irregularis), inasmuch as several superficial acts of breathing follow each (resp. sublimis, superficialis), then one very different, a deep full inspiration; resp. profunda, and almost suspiriosa besides. On the whole, the interval of time between two inspirations is rather prolonged, so that the respiration is not frequent.

"This manner of breathing is distinct from other anomalous modes of respiration, is easily distinguished from them, and is always easily recognized. It is so characteristic, that it is known in semeiology as respiratio cephalica, a symptomatic individualized form and indication, to be known at once without hesitation. It is *a sign of diagnostic value*." (pp. 65-7.)

We have given the preceding as an example of our author's style. It is deficient in conciseness; is in fact conversational. This, however, we conceive to be rather an advantage than a defect, because it detains the current of ideas upon each symptom, and renders the impression on the mind more distinct and lasting. As our pages do not admit of diffuseness, we must compress the author's views and strip them of their wordiness.

This *respiratio cephalica* is in truth an important symptom in diagnosis. It is sometimes absent, it is true, but is generally found to accompany pressure on the brain from whatever cause. It is pathognomonic of pressure on the motor centres, whether induced by serous or purulent exudation, hemorrhage, heterologous formations, as fungus, tubercles, &c., or direct injury to the skull, causing depression. If observed in children suffering from arachnitis, it is a proof that exudation has already begun and advanced.

*Special diagnostic synthesis of the affection from empirical pathology.* Nothing is known of the previous history of the patient; no one can say anything about him, and he is unable to speak. It is true he is suffering from an affection of the brain, but what is the precise nature of the affection? It must have come on suddenly, and not long ago; his turgid, uncollapsed face shows that.

If it had come on at an earlier period, he would have been brought sooner to the hospital. It may be supposed to be apoplexia sanguinea, for the sudden hemiplegia, the loss of consciousness, and the character of the respiration point to this affection very distinctly. Opposed to this view, we have the increased temperature and the febrile pulse. Apoplexy from hemorrhage is characterized by a slow pulse and diminished temperature. If it be apoplexy there must be some complication, and the position of the head is in this respect a striking symptom. An unconscious apoplectic patient lying supine, never holds his head in a constrained position. And on examining the muscles of the neck they are found to be tense, swollen, and so tender to the touch, that if but slightly pressed the patient's countenance expresses pain, and he utters loudly some unintelligible words. Now rheumatism of the nuchal muscles not unfrequently extends to the vertebræ, and even to the spinal arachnoid membrane, inducing inflammation and its sequelæ. This, then, may be a cause of the accompanying fever.

In cases of apoplexy there is a stage in which the pulse becomes quickened and febrile symptoms arise; namely, the stage of softening. But these occur in from five to ten days after the attack, and the pulse is small and weak, as well as frequent. The face is also collapsed. We cannot infer softening, therefore, in this case. Sometimes a primary and sudden softening of the nervous centres is accompanied by hemiplegia; but it occurs either in very young or very old subjects, and principally the latter. There is also more consciousness than in this case, and the countenance is pale and sunken, the pulse weak.

As there is, therefore, cerebral hemorrhage, in what part of the brain has it occurred? Opinions differ widely as to the functions of the different parts of the brain, and as to the result of injury to different portions. Serres, Bouillaud, Foville, and others, have laid it down as a theoretical principle that injury of the thalami induces paralysis of the upper extremities; injury of the corpora striata paralysis of the lower extremities. As these parts decussate, the seat of the extravasation in the case before us must be referred (if we adopt Serres' views) to the right thalamus and its contiguous corpus striatum. The numerical method has shown that in about half the cases of apoplexy, the thalamus first, and then, with an increasing hemorrhage, the corpus striatum and the parietes of the ventricles are implicated. Hemorrhage on the surface of the brain is very rare in adults; hemorrhage at the base of the brain determines that rapidly fatal and incurable form termed by French writers apoplexie foudroyante. It is inferred, then, that the seat of the hemorrhage is at the points indicated.

*Physiological and practical analysis of the symptoms, with reference to the seat of the hemorrhage.* Having derived from experience, or empirical pathology, what information we can, it remains to examine the symptoms in their physiological relations. The physiology of the brain is so imperfect, and the views regarding its structure, relations to the nerves, and the functions of its various parts, so discordant and contradictory, that little can be done by this method. We will follow our author, however, through his attempted analysis; and, firstly, we have to consider the mental disturbance—the suspension of consciousness. Since the gray substance of the hemispheres is considered as the seat of the mental functions, we have in this case an impression radiated from the corpus striatum and thalamus to the hemispheres. Secondly, the activity of the senses is abolished. This may depend upon the loss of power in the hemispheres themselves; but we have proof, in the insensibility of the pupils to light, that the function of the visual centre is affected, for the reflex action through the ciliary nerves is interrupted. This fact does not, however, point exclusively to the thalami as the seat of the extravasation, because, in affections of the corpora quadrigemina and geniculata, and other deep portions of the hemispheres, these seem connected with the sense of vision. It is also certain that this insensibility of the pupils is sometimes sympathetic, and the result of an injurious influence radiating on the visual centres.

Thirdly, the nerves, both of motion and sensation, are affected, though in different degrees. Indeed as all nerves, whether sensitive, motor, or sympathetic, end in the brain, we may expect that all will be affected in diseases of the brain, as in fact they are. We will take the nerves in order. *a.* The Facial nerves. These seem unaffected in this case; there is no dragging of the face to one side. In many similar examples this symptom occurs. It is to be noted, however, that it is often but of short duration; often in a few hours after the fit, the lateral deviation of the facial muscles disappears. Hence it is to be supposed that it is sympathetic and dependent on a radiated influence. It is usually on the same side as the extravasation; sometimes, however, on the opposite—a circumstance not yet elucidated by physiology. *b.* The Spinal nerves. The paralysis of the left arm and leg has been already noticed. It is worthy of remark that in most cases the leg regains its power of motion more quickly than the arm, and that the function of the nerves of sensation is less affected than that of the motor nerves. Dr. Neisser thinks this fact proves that the central axis of the sensitive nerves is not the same as that of the motor. He is of opinion that they end in the medulla oblongata, and that the disturbance of their function is sympathetic and dependent on a radiated influence. But even the motor nerves may be unequally influenced. A young man was placed under Schönlein's care, in the clinical hospital at Zurich, with incomplete hemiplegia of the left side, and lateral deviation of the facial muscles. The attack was acute; he had headache, &c., and it was pronounced to be a case of cerebral hemorrhage with congestion. A few days after, Professor Arnold was examining the affected muscles more minutely, and found that while the brachialis anticus was paralysed, the biceps could be freely moved. Now the two muscles are supplied with branches from the same nerve, and, therefore, Professor Arnold urged that it was a peripheral, and not a central or cerebral affection. All the symptoms of the case were, however, opposed to this view, as well as the known fact that individual nerves, and they only, may be affected in diseases of the brain. The respiratory nerves are derived from the medulla oblongata, and their origin is, therefore, far distant from the seat of extravasation. We, however, find them affected in the case under examination; and the probable conclusion is, that they suffer in the same way as the hemispheres, namely, by irradiation. It is worthy of special notice that the respiratory muscles in this case are hemiplegic; for the left side of the thorax is motionless. *c.* The Sympathetic or Organic nerves participate in the sensorial disturbance; the bowels are constipated; the urinary bladder becomes distended; and the activity of the cardiac movements is diminished. Whenever the brain suffers from compression, the pulse becomes slower; the more indeed the former is compressed, the more the latter is retarded, becoming at the same time larger and fuller. This is the pulsus cephalicus of the ancients. To the experienced practitioner the condition of the pulse is of great importance in the diagnosis. It gives often the first warning of commencing cerebral disease. In those cases in which the hydropical effusion takes place slowly and insidiously, without any symptoms referrible to the head, the retardation of the pulse is the first symptom that indicates the impending danger.

*Etiology of the affection.* What is the cause of the apoplexy in this case? It cannot be ossification of the cerebral arteries, or softening of the brain, or the apoplectic predisposition. These belong to advancing years. Young individuals may have apoplexy from two causes; namely, hypertrophy of the left ventricle, and ectasis or dilatation of the cerebral arteries.

*Ectasis vasorum cerebri* is a form of disease of which little information has hitherto been gathered by systematic writers. Dr. Neisser has seen it in two young men, aged from 20 to 30, and in a female with amenorrhea, aged 19—all characterised by a plethoric habit, short neck, thick head, stunted growth, &c. The disease most frequently attacks the basilar artery and its branches. These are simply dilated equally along their whole length, and not in aneurismal pouches. The blood moves, consequently, more slowly along them; in fact, the disease is not easily distinguished in its early stage from active congestion dependent on other causes. The patient is readily heated, complains of overflow of blood, headache, sensual disorder. These symptoms recur continually, in spite of energetic and methodical treatment; and at length become inveterate, and excite anxiety. The patient complains at last of vertigo, and a peculiar heaviness and dulness of the feet. He has neither pain nor formication, but walks with a tottering, uncertain step, staggering first to one side, then to the other, as if intoxicated. He is often so affected with vertigo that he falls, particularly towards evening, and in the dark. All the symptoms are aggravated at night, and remit in the morning. There are partial sweats on the head and face, and in some cases amblyopic symptoms. Dr. Neisser has found an energetic antiphlogistic and revulsive method perseveringly carried out, and under favorable circumstances, to be entirely inefficacious. Death is generally sudden. There is no reason to presume the existence of either hypertrophy of the left ventricle, or ectasis of the basilar artery, in the case under consideration. The probability is, that the extravasation is connected with the rheumatism of the nuchal muscles, and that there has been a metastasis to the brain or membranes.

*Prognosis of the case.* The impression on first looking at the patient is unfavorable. The intensity and duration of the stage of unconsciousness is, perhaps, the best prognostic sign. If an apoplectic patient lies utterly insensible to all stimuli, breathing stertorously, with open mouth and turgid cheeks, passing stool and urine unconsciously, &c., death is inevitable. On the other hand, a transitory loss of consciousness, with slight paralysis, by no means augurs an unfavorable termination. It is in the mixed cases that the greatest uncertainty arises. Here the patient answers, although unintelligibly, when spoken to. Neither is he worse than he was twenty-four hours ago. So far the prognosis is favorable; but the inflammatory action is a serious complication, and renders the termination doubtful.

*Treatment.* Venesection to 12 ounces; ice to the head; internally, nitre with sulphate of potass. The bleeding to be repeated, if the symptoms are aggravated.

Friday, 17th. Let us accompany Dr. Neisser on his visit to his patient, and see what has been the result of the treatment. Bleeding was performed forthwith; the blood drawn had a firm clot, with two teaspoonfuls of serum. Immediately after the bleeding the patient opened his eyes; the pupils acted, and the paralysis of the left arm disappeared. A paralysis to disappear in two days! Was there really, then, extravasation of blood, or was the diagnosis of yesterday wrong, and there was only extreme cerebral congestion? Was it an apoplexia intra vasa, a paraplexia, a coup de sang? No; the paralysis is opposed to this opinion. In all forms of paraplexy, whether in plethoric young persons, or occurring as apoplectic molimina, there is very seldom, *if ever*, hemiplegia or complete paralysis. But the readiness with which the paralytic affection has disappeared points out another locality for the extravasation than that fixed on yesterday. Cases of sanguineous apoplexy are dangerous in proportion as the locality of the hemorrhage is distant from the surface of the brain. Hemorrhage at the base of the brain is the most dangerous and most fatal; hemorrhage on the surface of the convolutions is the least dangerous and least fatal. The latter occurs the most frequently in children, and constitutes the apoplexia neonatorum. The hemorrhage is usually consequent on exudation through the capillaries, or, according to Cruveilhier, from the smaller venous trunks—at least in children. In these examples, the apoplectic symptoms are much less dreaded, and more gradual, than in other forms. The symptoms of the case in hand are of this character: the paralysis has been easily removed, the loss of consciousness is incomplete; the extravasation is not into the right thalamus and corpus striatum, but on the surface of the left hemisphere.

After a clyster, the patient had three motions; retching and vomiting took place whenever he drank liquids; he was restless, and had no sleep during the night. To-day he tosses his head about, sometimes his hands, sometimes his legs; he answers questions hastily and imperfectly, in a sort of half-delirious manner. The face is red, the eyes injected, the head and cheeks hot, the pupils contracted. It is found, however, that the nuchal muscles are no longer tense, and the head is moved about much more freely, and without pain; but there is considerable pain on pressure in the region of the third, fourth, and fifth cervical vertebræ. The breathing is quite otherwise than yesterday; it is frequent, deep, regular; the vomiting continues; the urine is scanty, and strongly saturated; the skin is hot and dry; the pulse is 92, large, and strong, but its rhythm is irregular; a few beats go quicker than the preceding, and then the rhythm becomes slower again—a significant symptom; a symptom to be carefully noted in all cerebral affections. The symptoms are now altogether changed; the diagnosis is difficult, but the grounds must now be stated for pronouncing it a case of acute arachnitis at the base of the brain, with commencing exudation of pus, and complicated with meningitis spinalis in the same (or purulent) stage.

What are the reasons for this diagnosis? The leading symptoms indicate to-day a state of cerebral irritation; we need not repeat them, but they were masked yesterday by the pressure on the brain. There was no restlessness, no active delirium, no shouting; the brain was in a state of torpor. Venesection removed the cause of this, and the true characters of the disease appeared. Now that this state of irritation is inflammatory is manifest from the febrile symptoms, and that it is not encephalitis is probable, because that disease is rare; and, further, the rheumatic affection of the nuchal muscles indicates the serous membrane as the locality of the inflammation. It is to be noted, too, that a rheumatic affection of the nuchal muscles is a prominent circumstance in the natural history of arachnitis.

The nature of the inflammation is such that it must have continued three days; the period, then, for serous exudation is past; it is the period of puriform exudation. Systematic writers generally describe the two stages as being distinctly marked, the second being the stage of compression; but this, Dr. Neisser declares, is quite wrong. There is always a transition-stage which complicates the diagnosis much, because it complicates the symptoms. The symptoms of compression are at first mingled with those of irritation, and it is only near the fatal termination of the case that the latter disappear. In the case before us the sensibility of the pupils, the active delirium, the expression of pain, and the vomiting, are all marks of irritation. Vomiting, like the peculiar pulse, is a specially important symptom in arachnitis. It is usually preceded in children by a tendency to yawn and by nausea; when fully established it is easily excited by the ingestion of medicine or food. The matter vomited is generally a watery mucus, occasionally mixed with bile, and it is rejected without much effort. Andral limits the symptom to the first, or purely inflammatory, stage of arachnitis; Dr. Neisser has found it frequently present in the second stage. He, therefore, concludes that the vomiting of yesterday indicated that the arachnitis had already attained the second stage, and that morbid products were formed. The febrile symptoms then observed were dependent upon the active inflammation going on; the compression of the brain masked the general symptoms of arachnoid inflammation.

The prognosis is now most unfavorable; there is no hope for the patient except in active treatment; arachnitis, if not checked, invariably conducts to death. In proposing a repetition of the bleeding, Dr. Neisser makes several practical remarks on that operation, for which we regret we cannot find room. Schönlein thinks that if the product-stage has arrived, all remedial means only hasten on the fatal termination. Dr. Neisser thinks, however, that cures of arachnitis in the second stage are sufficiently numerous, and that Schönlein is wrong in his therapeutics. Andral, Abercrombie, Portal, &c., forbid venesection, but recommend leeches. Dr. Neisser thinks that no rule can be laid down, but that leeches only can be of but little use in so intense and dangerous a form of inflammation as acute arachnitis. It is certain that bleeding in the latter stages may hasten on dissolution; the pulse becomes suddenly weak and fluttering, and the patient sinks rapidly. A careful diagnosis will, however, indicate the cases in which general bleeding is inadmissible. In the present example, Dr. Neisser prescribed bleeding to eight ounces, twenty leeches to the neck, ice to the head, calomel and jalap to act on the bowels.

Saturday evening, the 18th. The patient's relatives have stated what happened to him. On Tuesday he complained of hemiplegia, and on Wednesday he fell into the unconscious condition in which he was brought to the hospital. This circumstance confirms the diagnosis that the form and site of the hemorrhage was as last indicated; namely, capillary exudation on the surface of the left ventricle. The palsy preceded the loss of sensibility, and came on gradually—an important distinction. The patient did not, as was expected, experience any evil results from the venesection. The pulse was less irregular; the vomiting had ceased. In other respects the symptoms varied little. There was excessive pain in the neck, but more composure of manner; sleeplessness during the night, and active delirium. We shall not follow Dr. Neisser through his estimate of the symptoms; but the result is a different diagnosis to that on the preceding. Now it is arachnitis of the cervical cord extending to the medulla oblongata and base of the brain; in the first or inflammatory stage, the prognosis is more hopeful, but doubtful, on account of the persistent sleeplessness. These opinions seem principally based on the greater rationality and mental composure of the patient, a state of things incompatible with the more advanced stage of the disease. The increased cardiac action is manifest in the exquisitely inflammatory pulse, and indicate the necessity of a repetition of the bleeding, which is ordered to the extent of one pound, but without leeches. We give the result verbatim:

"Sunday, 19th. We have a foreboding of evil as we approach the bed; there is an ambiguous character of repose and passiveness, an inexpressive eye. The pulse hurries on at the rate of 130, small, and weak. It is all over; the patient is dying. It is the last stage, the stage of exhaustion, the herald and forerunner of inevitable death. . . . . . . After the bleeding there was a gradual abatement of the excitment. The pulse lost its tension, but remained strong and good, and the whole appearance and condition of the patient was satisfactory. An evening exacerbation was to be expected under the existing circumstances, and an increase in the symptoms. It came, was rather severe; so that bleeding to six ounces was again resorted to. A satisfactory remission followed, and an improved condition. The patient also fell into a quiet sleep, which continued for two hours. Towards morning he again became restless, but weakly, and without energy. He muttered deliriously, the pulse sank, became frequent, profuse diarrhea set in, and the stools were passed involuntarily.

"Such is the history of the circumstances that now appear before us. He lies in a constrained position, apathetic, muttering to himself, whether addressed or not, unintelligibly, inarticulately; no trace of consciousness; the eyes dull and staring; the temperature neither increased nor diminished; the pulse as described."

Such is the closing scene of the pathological drama which Dr. Neisser has written for the instruction of his readers. To them, at least, the catastrophe is in the post-mortem. It is as follows:

The dura mater was injected, the inner membrane dark red; much congested; could only be raised up in large patches, and with difficulty; and a quantity of reddish serum was collected beneath the arachnoid. There was an extravasation over nearly the whole of the convolutions of the left hemisphere, consisting of a thin layer of blood, partly coagulated and partly fluid, and dark. Both the pia mater and the compressed convolutions were at this point much congested, but no arterial or venous lesion was perceptible. The cortical substance was very vascular, and the medullary showed numerous red points. The parietes of the lateral ventricles, the thalami, &c., were all healthy; the ventricles contained some bloody serum. At the base of the brain, and particularly about the medulla oblongata, and the adjoining portion of the cerebellum, there was a thick, dirty yellow fluid, a purulent exudation between the arachnoid membrane and pia mater, the latter being much reddened and infiltrated with a watery fluid. In the dorsal region of the spinal cord there was a well-marked reddening of the membranes, which contained between them a collection of a yellow, thick, puriform fluid. The adjoining portions of the cord were affected, but the pia mater was much less reddened and infiltrated in the cervical region than in the dorsal.

*The Epikrisis.* We have now before us a tolerably complete statement of all particulars connected with this instructive case. It remains to institute an epikrisis. We must review the symptoms and see where the observer was right, where wrong. In the first place, the opinion as to the existence of a cerebral hemorrhage, and the judgment founded on the gradual accession of the paralysis and loss of consciousness, or the less degree of their intensity, and on their ready removal, as to the seat of the hemorrhage, are both confirmed by the inspection of the corpse. It is also apparent that the hemorrhage was not from an opening in a vessel (diabrosis), but was an exudation from the capillaries (diapedesis), consequent probably on the febrile excitement and inflammatory congestion. This mode of hemorrhage is also indicated by the *gradual* accession of the phenomena; if consequent on the rupture of a blood-vessel, the fit is more sudden and instantaneous. The paralysis was on the same side as the hemorrhage; this is an interesting circumstance, and is certainly an exception to the general rule. We do not yet know sufficiently the relations of the various parts of the brain to explain the differences. It is at least worthy of notice, that when there is decussating hemiplegia from hemorrhage into the thalamus or corpus striatum, it happens that the facial nerves are affected as often on the same side as on the opposite. Now the facial nerves take their origin above the decussation in the pyramids; why then are the facial muscles not subject to the same rule of paralysis in hemiplegia as those of the trunk?

The patient was restored to consciousness immediately after the bleeding; it could not have effected a removal of the exuded blood, because no bleeding could have effected that. It must then have been by relieving the congestion. Various considerations lead certainly to the opinion that pressure alone cannot always or indeed usually induce the unconscious state. It is more probably the "shock." We know that in cases of injury to the head, the brain becomes accustomed to considerable pressure without any aberration of function. So also in chronic arachnitis (hydrocephalus) and in diseases of the brain characterised by hypertrophy.

The condition of the pulse in different stages of the cases is worthy notice: on the first day it was 104, inflammation predominant; on the second day 94, and arhythmical—a mixed stage of inflammatory action and purulent deposit on the brain; on the third day 105, tense and regular—pure, intense inflammation; on the fourth and last day 130, and weak—the brain exhausted.

There was dorsal meningitis, as intense as the cerebral, yet the most prominent symptom did not exist; there was not the slightest affection of the spinal nerves. Probably the attack was inevitably fatal. We believe, however, that the treatment was bad, and that it hastened the mortal event. The bleeding was carried much too far. We are of opinion that if the learned doctor had kept his reasoning to himself, and left Nature to her own devices, there would have been a better chance for his patient. He will know better ere he is threescore. But we must pass on to the second case, of which we must make shorter work than the last.

"February 15th, 1839.—We come to the bedside of a young man who is unconscious, with his eyes closed, speechless, motionless, his head and neck spasmodically drawn to the left side. From time to time there is a spasmodic agitation of the hands and fingers. Respiration quick; pulse sunken, 144. *Peracute arachnitis at the base of the brain.* The inflammation is remarkably intense, and has attained already to considerable purulent formation. The disease has done its worst; it has exhausted the vital powers of the organism; the assistance of art is too late. It is the beginning of the end.

"The patient, a young man aged 19, of delicate constitution and weak muscularity, was brought last evening to the Charité Hospital, delirious and unconscious. We learn that already for several days he has been in a delirious unconscious state, and has had a rose eruption on the feet. A physician was called to him yesterday, who ordered venesection and leeches to the forehead, and sent him here. After his arrival last night, he was attacked with opisthotonos for two hours, and was delirious the whole night. He is girt with a broad padded belt, to which soft leather rings are attached to receive the hand; a circumstance that convinces us he must have been raving violently, as these means of restraint are only used here as the last extremity, and when danger is apprehended. . . . .

"Let the reader imagine a young man stretched on the back, his head turned to the left side, the eyes closed, the countenance placid, as if he were asleep. Only a loud frequent respiration and convulsive movements of the hands and fingers break at short intervals the singular stillness which impresses the mind. The extraordinary appearance of such a patient rivets the eyes, and shocks the senses as well as the feelings. The impression made by the morbid phenomena is so powerful, because the uncommon quietude, the death-like insensibility of the patient, together with the unchanging unnatural position of the head, contrast frightfully with the powerful convulsions and the hasty elevation of the hands; the patient appears inwardly fettered by some morbid power, and the soul of the spectator is filled with irrepressible sensations. . . . . He gives no answers to our questions, he takes no notice of us. . . . . The face exhibits no capillary congestion, nor anything particular; it is neither remarkably hot, nor red, nor pale; the tone of the facial muscles is normal; neither the lips nor teeth are spasmodically compressed; there is no distortion of the mouth, no spasm of the eyelid, no lateral deviation of the face. We open the eyes—the patient is insensible to light, but the pupils are normal, and contract. The nuchal muscles are not swollen; if, however, we attempt to straighten the neck or the head, there is an expression of pain on the countenance, and the head is restored to its unnatural position. The respiration is loud and feverishly quick, but rhythmical, full, and deep; no vomiting; abdomen soft and natural; region of the bladder free. The extremities extended, but not paralysed. . . . . We can say nothing of the secretions (except that there is a perspirable skin), for the patient passes urine and fluid stools involuntarily." (p. 109.)

We have not space to follow our author through his diagnostic analysis of the phenomena in this case, his prognosis, his etiology, or his physiological estimate of the symptoms, in which he discusses several points in cerebral physiology, but go on to

*The treatment of arachnitis by cold affusion and the actual cautery.* Of course the nature, intensity, and stage of the affection in this case show that it is hopeless, but Dr. Neisser takes occasion to discuss certain means most likely to be successful in arachnitis. These are two: the actual cautery and cold shower-bath.

The actual cautery has the appearance of being a most painful and severe remedy. But it is in fact quite the contrary. It is surprising to observe, when applied without previously acquainting the patient with the intent, how little pain it gives. Dr. Neisser observes, that he has seen even children endure it without a cry, or appearing conscious of its application. We have ourselves been astonished to hear no expressions of pain when the living flesh has been hissing and frizzling beneath the glowing iron. Dr. Neisser states, that he has known men in perfect possession of their natural sensibility describe cauterizations several inches long as causing simply a sensation as of warm water being applied. Its remedial power by no means consists in a sudden shock to the system, for this it does not effect. It is an active derivant and counter-irritant. Dr. Neisser has found the actual cautery to act like a charm in checking deep-seated inflammation and exciting absorption, or rather in affording an opportunity to the so-called vis medicatrix to come into play. In diseases of the bones and articulations, its utility is great. Dr. Neisser founds an opinion as to its *modus operandi* on the well-known tendency to metastasis, exhibited by inflammation of serous membranes. The artificial irritation determines a metastasis of the original inflammation to the part irritated. It is necessary, however, that there be a thickness to muscle, or of cellular tissue between the cauterized and the inflamed part; otherwise it will only aggravate the existing inflammation. Its application to the head, or to the elbow, knee, feet, and fingers is inadmissible. Nor if the fever be violent should it be used, as it will, under such circumstances, be more injurious as a direct irritant than useful as a counter-irritant. It ought not, therefore, to be used in cerebral affections accompanied with active delirium and great irritability.

The effects of the cold douche, or shower-bath, are remarkable. Applied to a patient who is unconscious and insensible (as in the example under consideration), its first effect is a powerfully shuddering impression. The patient appears terrified, is aroused for a moment from stupor, sees, hears, and sometimes speaks; he complains, is restless, &c. Children especially exhibit great anxiety, they shiver, their teeth chatter, they cry aloud, weep, &c. If repeated, the same phenomena again appear, and the surface becomes cold, the skin contracted (*cutis anserina*), and the blood seems concentrated in the heart and lungs. The respiration is hurried, the pulse sinks. If the patient be rubbed dry and carried to a warm bed, in a moment or two all these urgent symptoms gradually disappear; the pulse regains its tone, the skin becomes warm, but instead of its former burning heat, it is now moist and agreeable. The kidneys secrete urine profusely, and the patient feels so comfortable and so much relieved, that he sinks into a gentle slumber, and will often request a repetition of the douche.

The cold douche is, however, a most potent depressing remedy, and should be used with great caution. Dr. Neisser says, that if continued long enough, it will induce so great prostration in the strongest man, that reaction will not easily be brought about. The frequency of its repetition, and the quantity and temperature of the water are to be decided by circumstances. The greater the heat of the patient, the cooler may be the douche. For an adult, from three to eight pailfuls of water may be used; and for a child, the same number of pitcherfuls, continued from two to fifteen minutes, according to the strength of the patient and the effect produced. A child should be placed in a tub half filled with warm water. The skin, if it perspires, should be carefully wiped dry, and the chest and neck wrapped up in a napkin to prevent the cold water touching them. This precaution should never be omitted, but it is doubly necessary when the disease is complicated with a thoracic affection or tendency to tubercular deposit. A little water should be first sprinkled on the child's face, and then poured in a broad stream on its head; an interval of a few seconds should elapse between each affusion. When the operation is completed, the child should be quickly rubbed dry, wrapped in a warm blanket or flannel, and put to bed. Dr. Neisser thinks the practitioner should himself perform the operation, or, at least, personally superintend it, and see the patient shortly afterwards.

Moderate cold affusion is a useful remedy in many cerebral affections. In passive hyperæmia or venous oppletion, such as is met with in drunkards, in delirium tremens, in narcotic poisoning, in congestion consequent on exanthematic fevers, and in low fevers (febris nervosa stupida). It is also a very valuable remedy in irritable states of the brain with active congestion, and in the premonitory stage of apoplexy. It is strongly contraindicated, however, in all cases where the debility is great; it is, therefore, inadmissible in the last stage of arachnitis. The end of the first, and the transition stage to the second, are the periods when it is most likely to be useful in that disease. In mania, and in all cases in which erethism of the brain is intense, the slightest degree of cold affusion is strongly antiphlogistic, and may be used and continued, until that state of prostration is attained to which we have before alluded. Care, however, must be taken with this, as with all other lowering means, that it be not carried too far.

After this long digression (and we are bound to say that Dr. Neisser makes a much longer, for he discusses at length the physiological action of cold affusion) we will return to the case in hand. It is apparently hopeless; but the zealous practitioner is true to his life-saving *métier*—"while there is life there is hope," is the maxim most deeply impressed on his mind. To gain time in acute affections is a leading indication; to push on the clogging weary wheels of life is his main object, until mayhap the cycle of disease is completed, and the struggle ends in victory.

The treatment Dr. Neisser adopted in this case was cold affusion, and the actual cautery to the nucha; ethereal oil of mustard to the scalp; poultices sprinkled with camphorated spirit to the whole surface of the leg. The patient died on the 16th.

*Post-mortem examination.* The dura mater was sound; the vessels filled with fluid blood. On the convex surface of the brain the membranes had a red appearance, in consequence of injection of the vessel of the pia mater; a small quantity of reddish serum was effused between the latter and the arachnoid. The substance of the brain was firm; the ventricles containing serum, but not distended beneath the arachnoid membrane at the base of the brain; there was a layer of a whitish green puriform fluid, which covered the under surface of the anterior lobes of the brain, the fissura Sylvii, the olfactory nerves, together with the pons, medulla oblongata, and the adjoining surface of the cerebellum. The subjacent brain was reddened, but not softened; the pia mater was not adherent to it. The spinal arachnoid membrane was filled with a flocky serum; the posterior surface of the upper cervical portion of the cord was covered with a purulent exudation, like that found at the base of the brain, and (like it) contained between the pia mater and arachnoid, the former membrane being tumefied and infiltrated.

The leg was inflamed and infiltrated with pus and dark red serum.

*Complications of arachnitis.* The next case is interesting, as an example of arachnitis complicated with certain morbid changes in the brain. The patient was a confirmed drunkard, had already suffered from delirium tremens, and was sent to the hospital by the attending physician as labouring under that disease. The characteristics of the delirium, peculiar to the latter disease, were wanting, while the other symptoms pointed at an inflammatory affection, but so doubtfully, that it was manifest there was some complication necessary—some complication of the disease which ought to be unravelled. There are several complications: the first is arachnitis with inflammation of the cortical substance of the brain; this occurs in about one half the cases of arachnitis; it does not, however, alter the series of symptoms. Another complication of arachnitis is well-marked congestion of the brain. Congestion of the cortical substance is probably a complication of nearly every case of arachnitis, on account of the intimate and direct vascular connexion between the pia mater and the brain; but this again makes no change in the symptoms. It is quite otherwise with the much rarer complication of arachnitis, namely, congestion of the medullary substance. In these cases the symptoms of excitement are masked by the congestion until after bleeding, when active delirium sets in, and the patient is apparently worse, although the change is really due to the removal of the pressure.

There are examples of congestion of the capillaries complicating arachnitis; but there is also a venous congestion, a hyperæmia of the cerebral veins. This complication is not often alluded to by systematic writers, but it is an important one; it is usually seen as the sequel of all habitual congestive states of the brain, but is never so constant as after the abuse of alcoholic drinks. In death from delirium tremens, the venous sinuses are not only turgid with blood, but the veins generally even to the small branches in the pia mater are congested and turgid, often giving rise to a thin lymphatic exudation, which is to be distinguished from inflammatory exudation, and not confounded with it, as has been done by some writers. A similar exudation takes place to a more or less extent in all parts of the brain as well as into the ventricles. The symptoms in the case under consideration tally with those which might be expected to occur in such a pathological state, but with this difference, that an actively inflammatory condition has aggravated the state of congestion, while the symptoms of the former have been masked by the latter. The diagnosis then is purulent arachnitis at the base of the brain, with serous effusion into the ventricles, caused by hyperæmia of the brain.

The prognosis was of course in such a case highly unfavorable. The treatment was as follows: 20 leeches were ordered to be applied to the head, and four grains of calomel and ten of jalap every three or four hours; on the next day 15 more leeches, and the cold affusion carefully applied; on the third day of treatment the symptoms became more threatening, and the patient gradually sunk until the evening of the seventh, when he died comatose. We will now proceed to detail the post-mortem examination, and see if the diagnosis was correct.

*Post-mortem appearances in the brain of a drunkard dead of arachnitis.* A quantity of clear serum was found effused between the two serous membranes; these were quite distended with serum, the arachnoid having a bladder-like appearance, besides being full of blood, in consequence of the highly congested and distended state of the veins over the whole convex surface of the hemispheres. Between the pia mater and arachnoid, besides a clear serum, there were found delicate fibrinous coagula in the form of narrow and broad threads adherent to the veins. Some of the venous trunks had a true varicose appearance. The cortical substance of the hemispheres was dark red, but otherwise healthy; so also the first slices through the medullary substance. On going deeper, however, the corpus callosum, with the adjacent septum pellucidum, and fornix were found much softened, and fell under the touch into a pulpy mass of a dull tinge, neither yellow nor red. The lateral ventricles were enormously distended with serum. The corpora striata and thalami were normal. At the base of the brain the arachnoid membrane had a dull pearly appearance, and beneath it a quantity of cloudy flocculent serum was collected; upon the medulla oblongata and cerebellum there was a quantity of milky puriform fluid. The spinal arachnoid membrane in the dorsal and lumbar regions was distended with a flocculent cloudy serum. At two points of the spinal cord, in the cervical and lumbar regions, the pia mater was injected, and the corresponding portion of the cord was softened, so that the knife passed through it as if it were gelatinous. The liver had undergone the fatty degeneration; the mucous coat of the stomach was softened; the small intestines exhibited slight intussusception, which were however easily unravelled by the finger.

It is to be remarked in this case, that there was softening of the brain and spinal cord; a condition not indicated by the symptoms. Dr. Neisser observes, that in this respect it is analogous to one half the cases of hydrocephalus, in which proportion there is a similar softening of the central parts of the brain, without any corresponding symptoms during life. Dr. Neisser supposes that this morbid change occurred a day or two before death, during the period of collapse.

The next case is one of a different character to any of the preceding. It is that of a journeyman weaver, aged 46, admitted into the hospital on July 31st, 1839. Dr. Neisser describes his appearance thus:

"His complexion and appearance is that of health; his expression intelligent and vivacious. Left to himself, there is nothing in his position, demeanour, or expression which would attract the observer's attention. To our inquiry as to what he suffers, and how long he has been ill, he answers very quickly in a loud tone, just as if he had been accustomed to converse with a deaf person; but he hears perfectly well, indeed listens attentively, and what he says is perfectly intelligent and connected. He states, that about fourteen days ago, he was chilled while bathing, experienced rigors and heats, and had suffered from headache, and particularly from sleeplessness and restless nights; in other respects he is healthy. The hands are hotter than natural, dry and rough. This, in conjunction with the state of the pulse (96-100), shows a certain amount of general febrile action. Tongue, thorax, and abdomen offer no symptoms." (p. 305.)

Dr. Neisser remarks on this case, that it is a remarkable example of disease in this respect, that only two or three days remain to elapse before the patient will be on the brink of a precipice, and yet he has almost all the appearances of health. The inexperienced practitioner would possibly consider it (recollecting the alleged cause) as a rheumatic fever, and would treat it with diaphoretics. He would hasten the fatal termination, and would be praised for his unremitting attention.

The peculiarities that distinguish this case from the preceding are the exalted sensibility of the sensorium, the quickness of the sensual perceptions, and the hastiness of manner. These, together with the other symptoms, point out a cerebral affection differing considerably from those previously narrated. On a more minute examination it is seen that there is some little injection and swelling of the eyelids; this symptom is significant, it indicates congestion of the head of some duration. There is also dilatation of the pupils, which continues even when the patient is opposite to a strong light. When the patient is raised and made to sit up, he complains of vertigo, and experiences heaviness and confusion of the head, so that he cannot keep himself up. There is, however, no tendency to vomiting. There is no symptom of paralysis in any of the limbs; the breathing is deep and regular.

The symptoms manifestly point to an inflammatory affection of the encephalon. The inflammation is not, however, peracute, having already existed 14 days; it will not terminate in the production of puriform exudation, for if the inflammation were of this character, death would already have ensued. It is therefore a subacute form, accompanied by and ending in a serous exudation. The increased temperature, the frequent and regular pulse, the regular breathing, the general irritability, all indicate the first stage of inflammatory excitement to be predominant. There are other symptoms which show that effusion has commenced, and the stage of oppression entered upon. The headache is replaced by vertigo and confusion of the head, experienced especially when the patient raises the latter. The dilatation of the pupils is a noticeable symptom, although Dr. Neisser thinks that too much pathognomonic importance has been attached to it. In fact, we are not yet certain under what circumstances the pupil contracts or dilates. In cases of this kind the effusion goes on for some time before it is indicated by well-marked symptoms. The brain (already excited) resists the oppressive effect of the effused fluid longer than it would if in its normal condition, so that the symptoms are not in proportion to the effusion. There is, indeed, a sort of interval, a point of counterpoise between the two agencies, which may be detected by the equal balance between the two classes of symptoms.

*Prognosis.* If the disease be left to take its course, and the patient be abandoned to the healing powers of nature, he dies. The vis medicatrix is here a fiction. The pulse is weak, yet the patient talks strongly, breathes freely, moves actively, has no expression of debility about him. There would appear to be a contradiction here ; nevertheless, the pulse is to be taken as a true index that the stage of exhaustion is not far off. This circumstance renders the prognosis unfavorable, and the more unfavorable, because the means necessary for the removal of the disease are such as will still further reduce the powers of the patient.

*Treatment.* The disease being still in the inflammatory stage, no absorption of the effused serum can take place; on the contrary, the quantity is hourly increased. This inflammatory action cannot be removed without the loss of blood, and the question arises as to the mode; that is to say, whether by leeches or venesection. Now the weakness of the pulse forbids general bleeding. In no class of diseases is this remedy so dangerous as in meningeal inflammation. Dr. Neisser explains it physiologically after this manner. Every period of activity continued and prolonged, without the interruption of an antagonistic period of repose, must necessarily lead to exhaustion. Repose is necessary to the repair of the vital mechanism. In meningeal inflammations the patient (that is, his brain) is deprived of this necessary repose, as in the case under notice. For this and other reasons which will occur to the reader, bleeding from the arm is not advisable. The treatment, then, is ten leeches to the temples (five to each) toward evening, to anticipate the evening's exacerbations; ice-cold applications, but no counter-irritants, as these will only add to the existing irritation. Action on the bowels and kidneys should be induced; and for this purpose a tablespoonful of the following mixture is to be taken every two hours. We give the prescription verbatim : " ℞. Magnes. sulphur, ℥ss. ; S. in aq. comm. ℥v. Adde—Oxym. squill, ℥j ; succ. liquor, ʒiij, M." The result of this method we will also state in the author's own words :

"Thursday, August 1st. In spite of the leeches and the ice-cold applications, there was febrile exacerbation, an increased heat of skin, and a pulse at 112, but no perceptible result of increased congestion. About ten o'clock the patient became very vivacious, sang a good deal, spoke much and loudly, then slept till one o'clock, when he awoke, and began to sing again. There have been four watery stools, but little urine passed.

"To-day the pulse is 92. When addressed the patient answers in good spirits; 'he is perfectly well, ails nothing at all, has no pain, and will soon be well,' &c.; and while he is thus talking, the pulse becomes stronger, hard, more frequent; but so soon as he is quiet the pulse falls again. Nevertheless, he is not worse than yesterday, but somewhat better." (p. 318.)

Dr. Neisser infers from the nocturnal exacerbation and the state of the pulse, that there is yet considerable cerebral action, and orders ten more leeches. The bowels having been well moved, he would now act on the kidneys by means of squills, as in the following formula : " ℞. Acet. squill. ℥ij ; kali carb. q. s. ad perf. saturat. Cui adde Aq. font. ; syr. alth. ana, ℥j."

This mixture, we presume, was taken as the previous one.

In the evening no exacerbation took place, the pulse fell to 50, with a good night and several hours of sleep. On the day following, the pulse at 60, the patient lay slumbering, and in breathing puffed his lips and cheeks; a serious symptom, as indicative of incipient paralysis. Cold affusion was now ordered instead of ice: a large blister to the nape, and an aperient. In the evening of this day vomiting took place twice, and the patient slept a couple of hours only.

On August 3d, the symptoms were more threatening; the patient puffed his cheeks still in breathing, the pulse only 52, the respiration cephalic; that dangerous form already noticed. The patient is less vivacious, but the intellect is little affected, and the disturbance being trifling in comparison with the other symptoms of compression, it indicates the effusion to be rather in the periphery than the base of the brain. The douche to be repeated, the blister kept open, the bowels and kidneys to be more stimulated. As the stomach is irritable, the squill must be omitted; calomel and jalap are, for this cause, inadmissible also; the following is a better dose: ℞. Tinct. colocynth, ℈ij; Aq. font. ℥iij; Syr. de spin. cervin. ℥j; a tablespoonful to be taken every two hours, according to circumstances.

From this period the patient began to recover. On a slight recurrence of the symptoms of compression, the following epispastic was sedulously applied to the head. ℞. Olei sinap. æther.; gtt. xxx; Spir. vini rect. ℥j. M. The urgent symptoms gradually subsided, and in eight days the patient was convalescent.

*Hydrocephalus acutus* is the next case. The patient was a lithographic printer, aged 37. His symptoms were headache and darting pains through the head; weariness, loss of appetite. The complexion is muddy, the expression thoughtful and anxious; when raised up he could scarcely support his head; was affected with nausea, vertigo, and little or no fever; pulse 72, irregular; skin dry, urine scanty and high-coloured. The patient reeled and tottered as he walked, so much so that he would have fallen unless supported: this is an important symptom, because indicative of pressure on the brain. The symptoms enumerated constitute the prodromi of acute hydrocephalus. The disease is frequent in adults, and not confined to children exclusively, as has often been stated. In the latter, in addition to the symptoms enumerated, there is often tenderness of the abdomen, which, in conjunction with the vomiting, sometimes leads to an erroneous diagnosis. Dr. Neisser thinks the first stage of hydrocephalus consists in general meningeal congestion: in the next stage, inflammation sets in, and the subsequent stages are as in arachnitis. It frequently happens, that the effusion takes place suddenly and with great rapidity.

Dr. Neisser began the treatment in this case with a general bleeding to sixteen ounces; purgation with compound infusion of senna; ice to the head, and a warm bath, temperature not higher than 26° Reaumur (90° Fahr.), the patient wearing an ice-cap while in it. After the bleeding, &c., the irregularity in the pulse disappeared, with the tendency to vomit. The bowels were freely moved, but the bath had no effect on the skin. Towards evening the patient complained of excessive pain in all his limbs, but striking particularly from the head through the neck and back to the feet, whenever he made the slightest attempt to sit up, or to move the head from one side to the other. There was considerable fever; the pulse 120, and active delirium, so that it was necessary to restrain the patient. In the morning the pulse fell to 84, the delirium and pain had ceased, and nothing remained but a sense of soreness in the nuchal muscles. There was no tenderness to the touch. Dr. Neisser attributes the apparent exacerbation of the symptoms to the removal of pressure from the brain by the bleeding and other measures, and thus allowing the results of the cerebral irritation (previously masked by the compression) to show themselves. The pain in the back and feet was dependent on irritation from congestion of the spinal meninges. Remedial means: cupping to seven or eight ounces (fifteen cupping glasses), then a bath, and if an exacerbation comes on at eight, a small general bleeding. Internally to take nitre and tartar emetic. The day following, the pain of the neck had gone, the pulse was 72, skin normal, mind composed. The previous night had been restless. The treatment was expectant: on the evening of the third day, symptoms of collapse began to show themselves; there was also a return of the pain in all the limbs, and in the neck and back; and that he might not increase it, he remained motionless. On attempting to raise him, he complained much, but there was no spinal tenderness. The pulse was 72, and little fever. Dr. Neisser now noted the case, as hydrocephalus complicated with arachnitis spinalis. It is worthy remark, that this apparent irregularity in the course and development of that disease, is in fact its normal course. Dr. Neisser now prescribed a bath with three pounds of carbonate of potass, and the following mixture: ℞. Liq. kali acet ʒj; Oxym. scill. ℥ij. Aq. font. ℥iij: a tablespoonful every two hours. The following ointment as a counter-irritant to the spine: ℞. Ung. mercur. —Ung. antim. tart. ana ℥ss. M.

From this period the patient began to recover, and was dismissed convalescent on the twelfth day of treatment.

The next, or 6th case, is one of arachnitis with effusion, four weeks after a fall from a stool. The 7th case is one of arachnitis spinalis with serous effusion, inducing paraplegia. We regret that our extended analysis of the preceding histories of disease renders any notice of these impossible. We will therefore pass on to the epilegomena, and cull a few practical or other interesting remarks.

*General notanda of arachnitis.* The first stage of an acute arachnitis is characterised by developed cerebral function in consequence of irritation; when the inflammation is passing into suppuration or exudation of pus, the symptoms of irritation are at first mixed up with symptoms of pressure on the brain: they then gradually disappear, and at last the latter only remain.

A difference in the degree of inflammatory action determines the nature of the inflammatory product: the subacute is characterised by serous exudation, the peracute by purulent. The subacute form is more gradual and imperceptible in its course to a fatal termination; in the peracute the successive stages of the disease rapidly follow each other, and the symptoms of cerebral excitement and the general inflammatory reaction in the system is more strongly developed. The earlier stages of the subacute disease are often insidious, and considerable effusion takes place before there is any very manifest disturbance of cerebral function. Occasionally, indeed, when the inflammatory action is both subacute and circumscribed, symptoms of pressure on the brain are the first indicants of the disease. If, on the other hand, the inflammation be general, but of low intensity, it constitutes another distinct and well-marked variety of arachnitis, known as the premonitory stage of acute hydrocephalus.

The complications of arachnitis are—1. With venous hyperæmia of the brain. This is usually seen in drunkards, and leads to a mixed form, in which there is neither an exclusively serous nor puriform exudation, but a combination of the two. If, however, the inflammation thus complicated be peracute, that dangerous form of disease is developed, known as apoplexia serosa. A second complication of arachnitis is with hemorrhagic apoplexy, or, in other words, an extravasation of blood into the substance, or on the surface of the brain. In this case, the symptoms of irritation are repressed by the pressure on the brain, and are only developed when the cause of that pressure is removed. A third complication of arachnitis is congestion of the brain. In this the symptoms vary according to the degree of congestion, and the case is most intense; they are analogous to those of arachnitis complicated with serous apoplexy. These general views are applicable also to spinal arachnitis.

The physiological inferences deducible from these cases of arachnitis are various. Firstly, it is shown that the symptoms of pressure on the brain are not urgent in proportion to the amount of extravasated blood or effused fluid, but have a relation to the rapidity or suddenness with which it occurs. This is a general principle, applicable to all agents injuriously acting on the brain, as moral impressions, &c. Secondly, there is a greater loss of consciousness when the inflammation and its product are situate at the base of the brain than when on the convex or lateral surfaces. It is thus also with cerebral hemorrhages; hemorrhage at the base induces the most profound and incurable coma; hemorrhage into or near the ventricles induces a less profound and more curable coma; the coma from hemorrhage on the periphery is the lightest and the most manageable of all. This general fact is capable of a satisfactory solution, if we adopt the doctrines of *reflex cerebral action;* and, so far from being opposed to the general opinion, that the mental acts originate in the hemispheres, may be made to harmonize with it. The incident excitor action, which excites the changes in the hemispheres necessary to mental acts, is cut off in its course from the periphery to the centre by the pressure on the conducting fibrils as they pass up from the cord along the base of the brain. A pressure here, indeed, induces a state of the hemispheres analogous to that in sleep; the stimulus from without is stopped. Now, when there is pressure from effusion on the hemispheres, it is only a counter-pressure that is exercised on the base of the brain; and so soon as this is alleviated, the channel of incident excitor impressions is again opened, and pressure is now only exercised on the upper convolutions, a considerable portion of which we know may be injured, and even removed, without any impairment of consciousness whatever. Coma, then, results rather from interruption of function, consequent on an interruption of the flow of the natural stimulus to the brain from the periphery, than on a derangement of structure; it is a result of pressure on the conducting media, rather than on the acting fibres. The same views will of course explain those phenomena (as irregular action of the heart, of the respiratory mechanism, &c.) which are dependent on an obstruction of the cerebral stimulus, in whatever that may consist; while the pressure on the conducting structures at the base interrupts the course of the incident excitor stimulus to the brain, it cuts off the supply of reflex cerebral stimulus to parts below. There is some consideration due, however, to the fact, that inflammations at the base of the brain are usually the most intense, and the extravasations the greatest, and consequently that the pressure under these circumstances will be greatest. But this circumstance is opposed to the well-known tolerance of injury which the hemispheres exhibit, so that, after all, the inference must be, that the greater the effusion, the greater the pressure on the conducting strands at the base of the brain.

To give the results more in detail. If there be apoplexy at the base of the brain, there is profound and persistent coma, paralysis of all the limbs, paraplegia, and generally a fatal termination. If there be inflammation at the base of the brain, violent spasms, namely, opisthotonos and trismus, followed by deep coma. If there be structural disease at the base, sensorial disturbance, great tendency to vertigo and syncope, titubation, &c.

Paralysis of the extremities may disappear, even when softening of the brain or spinal cord has taken place. A sufficient number of accurately observed cases have now established this principle, which sets aside the more current opinion, that in all cases in which paralysis or spasm is transient, there is *no* structural change in the nervous centres. At the same time, it cannot be denied, that in cases of arachnitis, such lesions of motility are sympathetic, and depend on an irradiation of morbid influence. It is a practical principle that spasm generally indicates a state of irritation in the nervous centres, and paralysis the contrary.

A circumstance worthy consideration is, that while the bowels are torpid in diseases of the brain, the stomach is irritable. Vomiting, as has been seen in the cases detailed, is an usual symptom in arachnitis. It generally accompanies concussion of the brain, and those chronic diseases of that organ which induce pressure. The physiological explanation is, that the vagus is affected at its roots by irradiation from the diseased portion; but then comes the question, why are the gastric branches only affected? Why, in short, is there cerebral vomiting, but not a cerebral cough? There must be some as yet unknown anatomical arrangement of the twigs making up the vagus.

The termination of the sympathetic nerves in the brain is demonstrated by pathology. The heart, intestinal canal, and urinary organs, all participate in the disturbance of the system caused by changes in the central axis. The pulse becomes irregular, the intestines torpid, the sphincter ani and bladder paralysed. The connexion between the brain and sympathetic system leads to remarkable modifications in the symptoms of certain diseases. Thus, in abdominal typhus, when active delirium sets in, the diarrhea, tenderness of the abdomen, &c., subside. If, in the course of a phthisis pulmonalis, cerebral disease occurs, and the patient becomes furiously delirious,—a complication we have occasionally observed, the cough, expectoration, and dyspnea disappear. We have heard a phthisical patient under these circumstances vociferate loudly, talk incessantly, be free from cough and from all rational signs whatever of disease of the lungs, when a post-mortem examination, made within forty-eight hours, has revealed the most extensive tubercular disorganization, so extensive, indeed, that there did not appear six inches square of healthy lung in the whole chest. In the same way we have observed extensive pneumonia to run through its course altogether unobserved and unsuspected by the practitioner in ordinary, until the stethoscope has demonstrated to him the presence of great and irremediable disease within the thorax. Again, we have observed a peracute gastritis to coexist with arachnitis: the stomach has been found after death so injected as to resemble a piece of red velvet, and yet during life there was not one symptom observed that could be traced to a morbid condition of that viscus.

Some of these complications may accelerate the fatal termination in arachnitis. The influence radiated on the pulmonary branches of the vagus may be so great as just to arrest irritation of the incident excitor nerves, and to prevent fits of coughing, but may not paralyse the organic nerves, and stop secretion. Then the mucus accumulates in the bronchi, and the patient dies at last suffocated. This state of things may not exist during the waking condition, but may, and indeed often does, supervene with sleep. In these cases sleep is dangerous, and, if the patient be not roused from it from time to time, will terminate in death.

Dr. Neisser insists that encephalitis never occurs except as the result of mechanical injuries, or as a complication of arachnitis, when it is confined to the portion of brain adjoining the inflamed membrane. The same remark applies to myelitis. But, under the term arachnitis, he means to describe a disease which has its true and primary seat in the pia mater, and which implicates the arachnoid membrane consecutively. The latter he looks upon as pathologically the cellular tissue of the pia mater. These membranes conjoined form the substratum of all idiopathic inflammation within the encephalon, while inflammation of the dura mater and of the substance of the brain follows only on mechanical injuries. We state these opinions doubtfully, and rather with the intent that our readers may know what is current among German pathologists, than for their adoption and approval. If the new views could be entertained which describe the arachnoid as a membraneous plexus of organic nerves, the pathology of the serous inflammations we have discussed must have a complete and minute reviewal. Possibly, indeed, there may be a clue obtained in this way to those inappreciable changes in the brain which give rise to delirium and other sensorial phenomena.

We have now to state in conclusion, that, after a careful perusal and analysis of Dr. Neisser's volume, we find it to be a valuable contribution to the natural history of disease. We cannot, indeed, recommend a more efficient plan than that which he adopts for the elucidation of obscure points in, and the general advancement of, pathology. The estimate of the symptoms, and their relations from the results of experience and the data of physiological science must be peculiarly rich in results. There is one great defect in Dr. Neisser's method, and that is, no attention is given to the etiological relations of the diseases he describes. We find no chemical analysis of the urine, no investigation into the condition of the blood, no consideration of the causal antecedents. The whole treatment is unmitigated empiricism.

